# Chemical Characterization and In Vitro Anti-Cancer Activities of a Hot Water Soluble Polysaccharide from Hulless Barley Grass

**DOI:** 10.3390/foods11050677

**Published:** 2022-02-25

**Authors:** Yijuan Xu, Chuangchuang Zhang, Meng Qi, Wuyang Huang, Zhongquan Sui, Harold Corke

**Affiliations:** 1 Department of Food Science & Technology, School of Agriculture and Biology, Shanghai Jiao Tong University, Shanghai 200240, China; xyj1005@sjtu.edu.cn (Y.X.); cczhang@sjtu.edu.cn (C.Z.); chirmons@foxmail.com (M.Q.); 2 Institute of Agro-Product Processing, Jiangsu Academy of Agricultural Sciences, Nanjing 210014, China; 3 Biotechnology and Food Engineering Program, Guangdong Technion-Israel Institute of Technology, Shantou 515063, China; harold.corke@gtiit.edu.cn; 4 Faculty of Biotechnology and Food Engineering, Technion–Israel Institute of Technology, Haifa 3200003, Israel

**Keywords:** hulless barley grass, water-soluble polysaccharides, chemical characterization, cell proliferation inhibition, anti-cancer

## Abstract

Hulless barley grass may confer many health benefits attributed to its bioactive functional components, such as polysaccharides. Here, a hot water soluble polysaccharide was extracted from hulless barley grass, and its chemical characterization and in vitro anti-cancer activities were investigated. The yield of hulless barley grass polysaccharide (HBGP) was 2.3%, and the purity reached 99.1% with a polydispersity index (PDI) of 1.11 after purification by a diethylaminoethyl cellulose (DE-32) column and an S-400 high resolution (HR) column. The molecular weight and number-average molecular weight of HBGP were 3.3 × 10^4^ and 2.9 × 10^4^ Da, respectively. The monosaccharide composition of HBGP included 35.1% galactose, 25.6% arabinose, 5.5% glucose, and 5.3% xylose. Based on infrared spectrum analysis, HBGP possessed pyranose and galactose residues. In addition, this water-soluble polysaccharide showed significant cell proliferation inhibitory effects against cancer cell lines HT29, Caco-2, 4T1, and CT26.WT in a dose-dependent manner, especially for HT29 (the half-inhibitory concentration IC_50_ value = 2.72 mg/mL). The results provide a basis for the development and utilization of hulless barley grass in functional foods to aid in preventing cancer.

## 1. Introduction

Hulless barley (*Hordeum vulgare* L. var. *nudum* Hook. f) is a cereal crop with nutritional and health benefits. Hulless barley is mainly cultivated in the Qinghai-Tibet Plateau and used in beverages, bakery products, and food supplements in China [1]. Hulless barley grain possesses unique physical and chemical properties and has good potential for numerous food and non-food applications [2]. Hulless barley grain is a good source of dietary fiber providing soluble and insoluble dietary fiber fractions, especially a much higher content of arabinoxylan and β-(1 → 3, 1 → 4)-glucan compared to hulled barley genotypes [3]. Recently, barley-related foods’ consumption decreased globally, except for beer brewing that uses barley as raw material for beverage production. However, there is a revival in barley grass due to the presence of nutritional components with benefits to human health.

The young green leaves and stem comprising hulless barley grass (HBG) also contain a useful range of functional components, providing further potential health-promoting benefits [4,5]. Hulless barley grass has been reported to possess many biological functions, including hypolipidemic [6], hypoglycemic [7], antiaging [8], anti-arthritis [9], antidepressant [10], anti-diabetes [11], anti-fatigue [12], anti-inflammation [13], antioxidant [14], and anticancer activities [15]. Daily consumption of HBG powder may help regulate blood sugar and blood pressure, facilitate weight loss, promote sleep, reduce gout and hyperuricemia, enhance immunity, improve gastrointestinal and liver function, alleviate atopic dermatitis, aid recovery from bone injury, and prevent chronic diseases, especially circulatory disorders [5,9,16,17]. With its high nutritional value and health benefits, HBG has attracted increased attention over the last decade and has the potential to become further commercialized for human consumption [18].

Hulless barley grass also has high dietary fiber content and demonstrates good antitumor effects. The increased barley grass consumption can help reduce cancer risk [19]. Dietary fiber cannot be digested and absorbed in the gastrointestinal tract, nor can it produce energy. Epidemiological studies have reported the protective effect of a fiber-rich diet against cancer, based on consumption of polysaccharides from various resources [20,21]. Previous studies reported that polysaccharides isolated from plants, fungi, and marine sources have been reported to inhibit a variety of cancer cell lines via different mechanisms, such as immunomodulation, suppression of reactive oxygen species, cell cycle arrest, disruption of the mitochondrial membrane, and nitric oxide production. Therefore, polysaccharides can potentially help inhibit some cancer cell types and help prevent metastasis [20,21,22,23,24,25]. The effectiveness of polysaccharides in health benefits is considered to be related to the fine structure of polysaccharides. For example, a purified acidic polysaccharide from *Sarcandra glabra* could act as a vaccine adjuvant to enhance anti-tumor effects of cancer vaccines [22]. Polysaccharides from *Cordyceps cicadae* could inhibit human cervical cancer Hela cells via apoptosis and cell cycle arrest [23]. Polysaccharide extracted from red alga *Jania rubens* showed in vitro anticancer activity against breast and colon cancer cell lines [24]. Polysaccharides may be regarded as suitable candidates for further investigation for therapeutics of cancer, such as colon cancer [25].

As the region with the largest daily consumption per capita of hulless barley, Yunnan province has the lowest cancer mortality. Shanghai has higher cancer mortality with markedly lower barley consumption [26]. In Japan, the recommended dietary intake for barely grass is 3–6 g. Green barley extract was reported to possess anti-proliferative and pro-apoptotic functions on leukemia and lymphoma as well as human breast cancer cells [15]. Hulless barley grass could inhibit the cancer cell growth attributed to the combined effects of the phytochemical mixtures. Potential anti-cancer components in barley grass including alkaloids, flavonoids, and chlorophyll have been reported [14,19]. The anticancer effects of polysaccharides against human cancer cells were widely investigated. However, the anti-cancer activities of polysaccharides from hulless barley grass have not been investigated as much as those of hulless barley grain. Most studies were focused on photochemical ingredients, such as flavonoids, chlorophyll, and polyphenols. In this study, a hot water soluble polysaccharide was extracted from hulless barley grass, and its chemical characterization and in vitro anti-cancer activities was investigated.

## 2. Materials and Methods

### 2.1. Materials

Hulless barley seed was bought from Qinghai Xinning Biological Technology Co., Ltd. (Qinghai, China). The seeds were treated to germinate and sprout before planting. Briefly, seeds were evenly spread on 2.0 cm thick nutrient soil after soaking overnight with distilled water in Shanghai Jiao Tong University Farm (Shanghai, China) in 2021. Then, the seeds were covered with a small amount of soil. After 14 to 21 days, the HBG was collected when the length of the barley leaves was about 15 cm. After freeze drying, the leaves were ground into power and passed through a 250 μm sieve. The samples were stored in closed 50 mL plastic tubes in a desiccator at 25 °C. The storage period was not longer than two weeks.

### 2.2. Chemical and Reagents

A mouse breast cancer cell line 4T1, mouse colon cancer cell line CT26.WT, human colon cancer cell line HT29, and human colonic carcinoma cell line Caco-2 were obtained from the Center for Excellence in Molecular Cell Science, the Chinese Academy of Sciences (Shanghai, China). Roswell Park Memorial Institute (RPMI) 1640 medium, Dulbecco’s modified Eagle medium (DMEM), and penicillin-streptomycin were procured from Thermo Fisher Scientific (Waltham, MA, USA). Fetal bovine serum (FBS) was procured from Gibco (Auckland, New Zealand). Dimethyl sulfoxide (DMSO) was procured from Sigma-Aldrich (St. Louis, MO, USA). An MTS (3-(4,5-dimethylthiazol-2-yl)-5-(3-carboxymethoxyphenyl)-2-(4-sulfophenyl)-2H-tetrazolium) cell proliferation colorimetric assay kit was purchased from Abcam (Cambridge, UK). Sephacryl S-400 HR was purchased from GE Healthcare Life Sciences (Pittsburgh, PA, USA). Cellulose DE-32 was purchased from Puyu Science and Trade Co., Ltd. (Shanghai, China). The enzymes α-amylase, papain, and glucoamylase, and the monosaccharide standards xylose, glucose, mannose, rhamnose, galactose, and arabinose were bought from Aladdin Bio-chemical Technology Co., Ltd. (Shanghai, China). All chemicals and reagents were analytical grade.

### 2.3. Extraction of Polysaccharide

Hulless barley grass power was defatted using a Soxhlet extraction with hexane. The extraction program was set up as follows: immersion at 55 °C for 90 min; extraction at 65 °C for 120 min; recovery at 75 °C for 15 min. Defatted HBG powder (10 g, d.b.) was extracted twice with 300 mL distilled water in a water bath at 80 °C for 3 h with constant stirring. Papain (0.15%, *w*/*w*) was added to hydrolyze and remove protein at 60 °C for 2 h. Then, thermostable α-amylase and amyloglucosidase were added to remove starch at 80 and 60 °C for 2 h, respectively. Sevag solution was prepared with a mixture of octanol and chloroform (2:1, *v*/*v*) was added to further remove protein and inactivate enzymes. The upper layer was collected after shaking vigorously in a separating funnel for 30 min. The upper layer of the solution was concentrated in a hot water bath at 80 °C until the volume reached to 100 mL. Three times the volume of ethanol (100%) were slowly added under constant rapid stirring at room temperature. The slurry was left at room temperature overnight, and then centrifuged at 1000**×* g* for 10 min. The precipitate was washed with acetone, dried under nitrogen, and labeled as hulless barley grass crude polysaccharide (HBGC).

### 2.4. Purification of Polysaccharide

As described by Li et al. [27], HBGC (300 mg, d.b.) was dissolved in distilled water (3 mL) and loaded into a diethylaminoethyl cellulose (DEAE-32) column (2.6 cm × 30 cm). The bed volume of DEAE-32 was 130 mL. The column was eluted with distilled water and gradient aqueous sodium chloride solution (0.25, 0.5, 1 mol/L) at a rate of 0.7 mL/min. Every 5 min, one tube was collected for a total of 80 tubes. After dialysis, the fractions were collected, concentrated, and further applied to an S-400 high resolution (HR) column (1.6 cm × 100 cm). The bed volume of S-400 HR was 190 mL. The column was eluted with distilled water at a rate of 0.2 mL/min and monitored again using the phenol-sulfuric acid method. The fraction was collected and lyophilized to make a purified polysaccharide, labelled as HBGP.

### 2.5. Determination of Purity and Molecular Weight

The determination of the purity of hulless barley grass polysaccharide was performed according to the method of Liu et al. [28]. HBGP (5 mg, d.b.) was dissolved in distilled water (1 mL) and passed through a filter (0.45 μm). The purity of HBGP was analyzed on a Waters Alliance e2695 High Performance Liquid Chromatography (HPLC) system equipped with a Waters 2414 differential detector and SUGAR KS-805 Shodex^®^ sugar column (8.0 mm ID × 300 mm L).

The molecular weight of HBGP was analyzed according to a previously described procedure [28]. The molecular weight of soluble HBGP was analyzed on an EcoSEC HLC-8320 GPC system (TOSOH Co., Tokyo, Japan), equipped with an EcoSEC refractive index detector. Dextran with different molecular weights (4400, 9900, 21,400, 43,500, 12,400, and 805,000 Da) was used to produce a standard curve to calculate the molecular weight of HBGP.

### 2.6. Monosaccharide Composition Analysis

Monosaccharide composition was conducted following a procedure described by Sui et al. [29] with minor modifications. Hulless barley grass purified polysaccharide (1 mg, d.b.) was hydrolyzed with 2 M TFA (0.5 mL) at 120 °C for 8 h with a Reacti-Vap^TM^ evaporator (Thermo Scientific, Rockford, IL, USA). The hydrolysate was evaporated to dryness with a nitrogen stream. The excess acid was removed by washing twice with isopropanol (0.25 mL). The residue was reduced by 0.5 mL of NaBH_4_ (20 mg/mL) containing 1 M NH_4_OH (0.1 mL) solution at 40 °C for 2 h, and then pre-acetylated with 1-methylimidazole (0.1 mL) and acetic anhydride (0.5 mL) for 15 min at room temperature. Alditol acetates were subjected to analysis using a 2010-plus gas chromatograph (Agilent Technologies, Santa Clara, CA, USA), attached to a capillary column DB-Wax (30 m × 0.25 mm id, film thickness: 0.25 μm; ChromPack, Middelburg, Netherlands) and a flame ionization detector. The column temperature was programmed as follows: initial holding at 100 °C for 2 min, then increased to 180 °C at 10 °C/min, held for 2 min, increased to 240 °C at 4 °C/min, held for 5 min, increased to 255 °C at 10 °C/min, and held for 5 min.

### 2.7. Fourier Transform Infrared (FT-IR) Spectra Analysis

The structural information of polysaccharide from hulless barley grass was carried out based on the method of Li et al. [27] with minor modifications. Hulless barley grass purified polysaccharide (10 mg) was mixed with potassium bromide powder and ground evenly to make a thin layer. The FT-IR spectra of HBGP were recorded using a Thermo Nicolet 6700 infrared spectrometer (Thermo Electron Corporation, Waltham, MA, USA), ranging from 4000 to 400 cm^−1^ at a resolution of 4 cm^−1^. The results were analyzed by OMNIC 8.2 (OMINIC software, Waltham, MA, USA).

### 2.8. Cell Culture and Treatments

Human colon cancer HT29 cells and Caco-2 cells were grown in DMEM containing 10% FBS and 1% penicillin-streptomycin, and mouse breast cancer 4T1 cells and colon cancer CT26.WT cells were cultured in RPMI 1640 medium supplemented with 10% FBS and 1% penicillin-streptomycin, kept at 37 °C and 5% CO_2_ in a Forma 371 CO_2_ atmosphere incubator (Thermo Scientific, Waltham, MA, USA). After reaching 80–90% confluence, the cells were sub-cultured. The cells were seeded in 96-well plates at concentrations of 5 × 10^3^ cells/well and incubated overnight to reach 50–60% confluence. The cells were treated with different concentrations of the HBGP (0.625, 1.25, 2.5, 5, and 10 mg/mL) and continued to co-culture for 48 h.

### 2.9. Cell Proliferation Assay

The cell viability was determined by an MTS cell proliferation colorimetric assay kit. A total of 100 μL of MTT (0.5156 mg/mL) was added to the cells treated with or without HBGP in 96-well plates and incubated at 37 °C and 5% CO_2_ for 4 h. After the MTT solution was removed, 100 μL of DMSO was added. Then, the mixture was shaken slowly for 10 min to dissolve the cell crystal. The absorbance at 570 nm was measured on a 1510 Multiscan GO microplate reader (Thermo Fisher Scientific, Waltham, MA, USA) to obtain the optical density (*OD*) values. The cells without HBGP treatment were used as the control, whereas wells without cells but containing the medium were used as the blank. The following formulas were used to determine cell viability and cell proliferation inhibitory rate:

Cell viability (%) = (*OD*_sample_ − *OD*_blank_)/(*OD*_control_ − *OD*_blank_) × 100%

Cell proliferation inhibitory rate (%) = [ 1 − (*OD*_sample_ − *OD*_blank_)/(*OD*_control_ − *OD*_blank_) ] × 100% (1)

### 2.10. Statistical Analysis

All the data were expressed as the mean ± standard deviation (SD) of six independent experiments. The figures were obtained using GraphPad Prism Version 8 (GraphPad Software, San Diego, CA, USA). One-way analysis of variance (ANOVA) with Tukey’s multiple comparisons test was conducted to determine statistical differences among different groups. *p* < 0.05 was considered to indicate significant differences.

## 3. Results

### 3.1. Isolation and Purification

**Figure 1:**
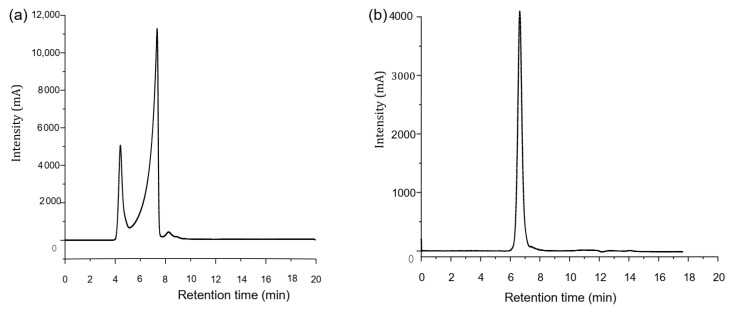
The purification of the hulless barley grass polysaccharide (HBGP). (**a**) HBGP purified by DE-32 column; (**b**) HBGP further purified by S-400 HR column.

The yield of hot water extracted polysaccharide from HBG was 2.29%. After loading into a diethylaminoethyl cellulose (DE-32) column, polysaccharide showed a doublet peak eluted with distilled water (Figure 1a). When polysaccharide was further purified with an S-400 HR column, HBGP presented as white power with a single, sharp, and symmetric peak in HPLC analysis (Figure 1b). It indicated that HBGP was successfully purified with DEAE and the S-400 column. The result showed that HBGP purity reached 99.1%. The structure of HBGP was homogeneous.

### 3.2. Molecular Weight of HBGP and Monosaccharide Composition

The molecular weight and number-average molecular weight of HBGP were 3.3 × 10^4^ and 2.9 × 10^4^ Da, respectively (Table 1). When the polydispersity index (PDI) value is close to 1, the corresponding monodispersed polysaccharide shows greater homogeneity [30]. The molecular weight was higher compared with the corresponding polysaccharides obtained from timothy grass, *Scutellaria barbata* grass, perennial ryegrass, and cocksfoot grass [31,32,33]. The PDI value of HBGP was 1.11, indicating a narrow distribution of polysaccharide with a relatively uniform structure.

**Figure 2:**
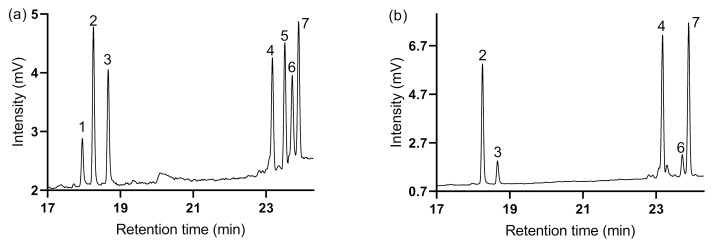
Determination of the monosaccharide composition of the hulless barley grass polysaccharide (HBGP). (**a**) Monosaccharide standards. (**b**) HBGP. (1), rhamnose; (2), arabinose; (3), xylose; (4), inositol; (5), mannose; (6), glucose; (7), galactose.

Based on the GC chromatogram of monosaccharide standards (Figure 2a), HBGP monosaccharide composition was determined (Figure 2b). Hulless barley grass polysaccharide is mainly composed of arabinose and galactose with a small amount of xylose and glucose. As shown in Table 2, the order of mass ratio of monosaccharide decreased as follows: galactose (35.1%) > arabinose (25.6%) > glucose (5.5%) > xylose (5.3%). The molar ratio of arabinose, xylose, glucose, and galactose content was 3.8:1.1:1:4.7. This indicated that galactose and arabinose are the dominant monosaccharides forming the backbone of HBGP.

### 3.3. FTIR-ATR Analysis

**Figure 3:**
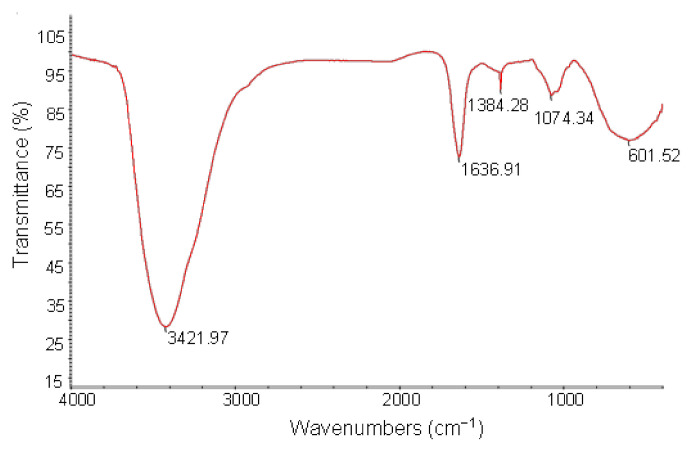
FTIR spectra of the hulless barley grass polysaccharide (HBGP).

The infrared spectrum analysis results of the water-soluble polysaccharides from the HBG are shown in Figure 3. The absorption peak at 3421.97 cm^−1^ showed the largest intensity and the broadest peak [34]. It might be caused by the stretching vibration of the OH bond, indicating that the molecular structure of HBGP might contain carboxyl group OH. The absorption peak at 1636.91 cm^−1^ is in the double bond stretching vibration region, which might be attributed to the stretching vibration of a C=C bond or a C=O bond [35]. Considering that the absorption peak is relatively strong, it is more likely due to the stretching vibration peak of the C=O bond. The absorption peak at 1384.28 cm^−1^ may be caused by the stretching vibration of -CH3. The absorption peak at 1074.34 cm^−1^ may be caused by the absorption vibration of the COC bond or the COH bond [27]. These results indicate that there were pyranose residues in HBGP. If combined with the monosaccharide composition analysis result, it is likely a characteristic peak of galactose residues.

### 3.4. Cancer Cell Proliferation Inhibitory Effects

**Figure 4:**
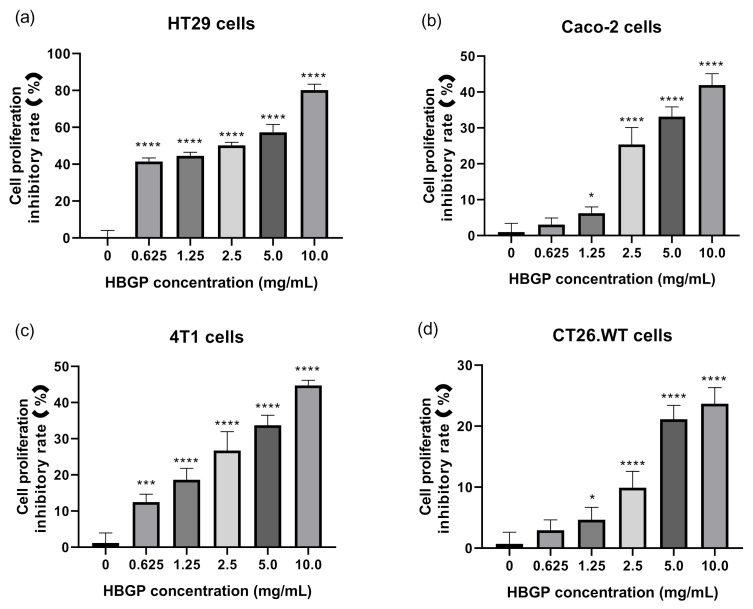
The inhibitory effects of different concentrations of the hulless barley grass polysaccharide (HBGP) on cancer cell proliferation for (**a**) HT29 cells, (**b**) Caco-2 cells, (**c**) 4T1 cells, and (**d**) CT26-WT cells. Bars represent mean values ± SD (*n* = 6). *, ***, and **** indicate *p* < 0.05, *p* < 0.001, and *p* < 0.0001 compared with the control NC (the concentration is 0 mg/mL), respectively.

The MTT method was used to test the cytotoxicity of water-soluble polysaccharide from hulless barley grass on HT29, Caco-2, 4T1, and CT26.WT cells. The decrease of cell viability explained that polysaccharides disturbed the cellular homeostasis and led to apoptosis. The results showed that HBGP had significant inhibitory effects against HT29, Caco-2, 4T1, and CT26-WT cell proliferation in a dose-dependent manner (Figure 4, *p* < 0.05) after 48 h treatment. When the HBGP reached a concentration of 10 mg/mL, the cell proliferation inhibitory rates of HBGP were 80.19 ± 3.18%, 41.94 ± 3.17%, 44.72 ± 1.44%, and 23.69 ± 2.62% for HT29, Caco-2, 4T1, and CT26-WT cells, respectively. Hulless barley grass polysaccharide exhibited stronger cytotoxicity to human cancer cells than to mouse cancer cells. The lowest inhibitory effect was found in mouse colon cancer CT26-WT cells, while a higher cell proliferation inhibitory effect was found in human colon cancer HT29 cells. As the concentration of HBGP was above 2.5 mg/mL, the cell proliferation inhibitory rate of HBGP was more than 50%, and its IC_50_ value was about 2.72 mg/mL according to the corresponding fitting curve (*y* = 4.0312 *x* + 39.055). Thus, this water-soluble polysaccharide from hulless barley grass could act as a potential anticancer substance.

## 4. Discussion

Hulless barley grass contains the same and different functional components as barley grains, supporting their similar and different health-promoting effects [4,5]. Hulless barley whole grains and their outer bran layer are rich in β-glucans, arabinoxylan, polyphenols, phenolic acids, flavonoids, phytosterols, alkylresorcinols, benzoxazinoids, lignans, tocols, folate, fiber, and resistant starch, which have antidiabetes, anticancer, antiobesity, antioxidant, antiproliferative, anti-cardiovascular disease, and cholesterol lowering abilities [4,36,37,38], whereas hulless barley grass is rich in gamma-aminobutyric acid (GABA), polyphenols, flavonoids, saponarin, lutonarin, superoxide dismutase (SOD), K, Ca, Se, tryptophan, chlorophyll, vitamins (A, B1, C, and E), metallothioneins, alkaloids, and dietary fiber, as well as polysaccharides, which have anti-diabetes, anti-arthritis, antigout, antioxidant, anti-inflammation, anti-acne/detoxifying, antidepressant, and anticancer effects [5,6,7,8,9,10,11,12,13,14,15]. Therefore, hulless barley grass, which is consumed as foods, i.e., a popular green-colored drink [16], is used in preventive treatment of chronic diseases, including cancer [9].

Hulless barley grass inhibits cancer cell growth by the combined effects of strong antioxidative properties, high alkaline contents, rich flavonoids, and chlorophyll [5,14]. The phytochemical mixtures of HBG exhibited a very good antitumor effect in female rats for the prevention of N-methyl-N-nitrosourea-induced mammary carcinogenesis and significantly decreased survival of breast cancer MCF-7 cells by preventing cell-cycle progression [19]. Green barley extract had anticancer activity by its selective anti-proliferative and pro-apoptotic functions on B-lineage leukemia/lymphoma cell lines as well as breast cancer cells of human beings, as manifested by G2/M phase arrest and DNA fragmentation [15]. HBG tricin could inhibit melanin biosynthesis in B16 melanoma cells, based on a hydroxyl group at the C-4′ position and methoxy groups at the C-3′,5′ positions of the tricin skeleton. Therefore it could be used as an antitumor agent against skin cancer [39]. Moreover, hulless barley grass can be served as a health food for dialysis patients at higher cancer risk, based on its absorbed exogenous functional ingredients applied from the outside [17]. To our knowledge, this is the first report to find out that polysaccharide from hulless barley grass is able to inhibit cancer cell growth, as it inhibited the cell proliferation of different cancer cell lines, including HT29, Caco-2, 4T1, and CT26.WT cells.

The physiological function of dietary fiber is closely related to its physical and chemical properties, especially its solubility in water [40]. Studies have shown that different soluble dietary fibers, such as beta-glucan, psyllium, pectin, glucomannan, and guar gum, can be used to reduce the risk of cardiovascular disease [41,42]. Despite large differences in molecular structure, no major differences exist between the different types of water-soluble fiber, suggesting a common underlying mechanism [41]. Dietary fiber is believed to decrease the incidence of colorectal cancer, but not all types of fiber are equally protective. Adsorption of carcinogens to insoluble dietary fiber in the intestinal tract is one of the mechanisms by which dietary fiber is believed to protect against colorectal cancer. However, soluble-fiber polysaccharides may enhance the development of colorectal cancer by reducing the ability of insoluble dietary fibers to adsorb hydrophobic carcinogens, crossing the intestinal epithelium and carrying with them carcinogens maintained in solution [43]. Interestingly, some water-soluble polysaccharides exhibited strong anti-cancer effects, such as in vitro anti-cancer active water-soluble polysaccharides from *Spirulina platensis* [44], and a water-soluble polysaccharide from the roots of *Polygala tenuifolia* suppressing ovarian tumor growth and angiogenesis in vivo [45]. Here, the water soluble polysaccharide extracted from hulless barley grass also exhibited in vitro anti-cancer activities.

The water-soluble polysaccharides extracted from hulless barley grass had different molecular weight and monosaccharide composition with those from barley grains, mainly β-glucans and arabinoxylan [4]. The water-soluble polysaccharide obtained from the grains of same species highland barley (*Hordeum vulgare* L.) had an average molecular weight of about 6.7 × 10^4^ Da and was composed of glucose, xylose, arabinose, and rhamnose with a relative molar ratio of 8.82:1.92:1.50:1.00 [46]. In this study, the average molecular weight of HBGP was a little lower than that from barley grains, and galactose and arabinose were the dominant monosaccharides of the water-soluble polysaccharide from hulless barley grass. Kim et al. [47] reported that the polysaccharide from barley grass included linear glucan (~10%), rhamnogalacturonan (30–35%), and glucuronoarabinoxylan (40–45%), based on monosaccharide composition and linkage analysis. Later, Kim et al. [48] claimed that the major polysaccharide in barley grass was arabinoxylan. The monosaccharide profile was related to: (1) biological varieties; (2) extraction methodology; (3) purification procedure; (4) analysis methodology [27,49,50]. The water-soluble polysaccharide obtained from barley grains with different molecular weight and monosaccharide composition was also found to inhibit proliferation of human colon cancer cells HT-29 in a time- and dose-dependent manner by inducing HT-29 apoptosis through ROS (reactive oxygen species)-JNK (c-Jun N-terminal kinase) and NF-B (nuclear factor-B)-mediated caspase pathways [46]. Here, HBGP exhibited similar cancer cell proliferation inhibitory effects. Therefore, polysaccharides from hulless barley grass could also provide a resource for functional food development with powerful anticancer activity.

## 5. Conclusions

In this study, a hot water soluble polysaccharide was prepared from hulless barley grass. This water-soluble polysaccharide was relatively uniform with galactose and arabinose as the dominant monosaccharides forming the backbone. After purification of DEAE and S-400 column, HBGP purity reached 99.1%. The molecular weight and number-average molecular weight of HBGP were 3.3 × 10^4^ and 2.9 × 10^4^ Da, respectively. The PDI value of HBGP was 1.11, indicating a relatively uniform structure. In the hulless barley grass polysaccharide, the molar ratio of arabinose, xylose, glucose, and galactose content was 3.8:1.1:1:4.7. In vitro anti-cancer activities confirmed that this polysaccharide could inhibit cell proliferation of various cancer cell lines, particularly human colon cancer HT29 cells. This study could provide a theoretical and scientific basis for the application of polysaccharides from hulless barley grass in functional foods and nutraceuticals to prevent cancer, and further study of the anti-cancer function and mechanism of hulless barley grass polysaccharides will be explored in the future.

## Figures and Tables

**Table 1:** The molecular weight of the hulless barley grass polysaccharide (HBGP).

Retention Time	Relative Peak Area (%)	Mn (Daltons)	Mw (Daltons)	Polydispersity Index
23.60	34.68	29,941	33,244	1.11

**Table 2:** Monosaccharide composition of the hulless barley grass polysaccharide (HBGP).

Peak	Retention Time	Area	Monosaccharide Composition	Area %
1	18.255	18,434.6	Arabinose	25.5918
2	18.665	3826.2	Xylose	5.3117
3	23.176	20,498.9	Inositol	28.4575
4	23.715	4004.6	Glucose	5.5594
5	23.889	25,268.9	Galactose	35.0796

## Data Availability

Not applicable.
